# TRACK & ACT: a pragmatic randomised controlled trial exploring the comparative effectiveness of pedometers and activity trackers for changing physical activity and sedentary behaviour in inactive individuals

**DOI:** 10.1186/s44167-023-00018-4

**Published:** 2023-05-01

**Authors:** Daniel J. Ryan, Megan H. Ross, Joshua Simmich, Norman Ng, Nicola W. Burton, Nick Gilson, Toby Pavey, Wendy J. Brown, Sjaan R. Gomersall

**Affiliations:** 1 School of Health and Rehabilitation Sciences, The University of Queensland, Brisbane, St Lucia, QLD 4072 Australia; 2 RECOVER Injury Research Centre, Faculty of Health and Behavioural Sciences, The University of Queensland, Brisbane, Australia; 3 School of Human Movement and Nutrition Sciences, The University of Queensland, Brisbane, Australia; 4 School of Applied Psychology, Griffith University, Brisbane, QLD 4122 Australia; 5 Menzies Heath Institute Queensland, Griffith University, Gold Coast, QLD 4222 Australia; 6 Centre for Mental Health, Griffith University, Brisbane, QLD 4122 Australia; 7 School of Exercise and Nutrition Sciences, Queensland University of Technology, Brisbane, Australia; 8 Faculty of Health Sciences and Medicine, Bond University, Gold Coast, Australia

**Keywords:** Physical activity, Sedentary behavior, Adults, Health-related behavior, Intervention, Activity trackers, Pedometer

## Abstract

**Background:**

Pedometers have been shown to be effective for increasing physical activity, however the potential additional effects of activity trackers, and their added capacity to simultaneously modify sedentary behaviour, has not been thoroughly explored. This study aimed to explore the comparative effectiveness of two activity trackers and a pedometer for improving daily step count and moderate-vigorous physical activity (MVPA), and reducing sedentary behaviour in inactive adults.

**Methods:**

48 inactive participants were allocated to one of three groups based on their workplace. Each group randomly received either a Fitbit ONE, Jawbone UP or Digi-Walker SW200 pedometer (PED) for 8-weeks and an orientation session to their respective device. Participants were informed about the study aims and were provided with their respective devices and where applicable, the associated Apps. Participants intentionally received no other active intervention components to simulate as closely as possible the experience of purchasing a device ‘off the shelf’. Step count, MVPA and time in sedentary behaviour were measured using accelerometry (Actigraph GT3X+) at baseline and four-, eight- and 16-weeks. Analyses were conducted using linear mixed-effect regression models to compare changes from baseline. Post-hoc tests of model estimates compared each activity tracker group to the pedometer group. Model estimates are reported for baseline-16 week follow-up.

**Results:**

At baseline, average (standard deviation) step count, MVPA and time spent sedentary was 6557 (2111) steps/day, 23 (13) minutes/day and 10.3 (1.0) hours/day in the PED group, 7156 (1496) steps/day, 26 (12) minutes/day and 9.3 (1.2) hours/day in the ONE group and 6853 (1281) steps/day, 29 (10) minutes/day and 10.1 (1.0) hours/day in the UP group. At 16-weeks, based on estimates from the linear mixed-effect regression model, the ONE and UP groups increased step count by 129 steps/day (95% CI − 1497, 1754) and 504 steps/day more (95% CI − 1120, 2130), respectively, than the PED group. For MVPA, the ONE and UP groups increased by 2.3 min/day (95% CI − 10.9, 15.4) and 2.7 min/day more (95% CI − 10.5, 15.8), respectively, than the PED group. For sedentary behaviour, the ONE group had 34 min/day more in time spent sedentary than the PED group (95% CI − 35, 104), while the UP group had 53 min/day more in time spent sedentary than the PED group (95% CI − 18, 123).

**Conclusions:**

All three groups demonstrated an increase in steps and MVPA, and a decrease in time spent in sedentary behaviour, however there was substantial individual variation in these outcomes indicating considerable uncertainty about the relative effectiveness of activity trackers and pedometers in improving PA and sedentary behaviour. Randomised controlled trials with adequate sample sizes are indicated.

**Trial registration:**

ACTRN12623000027617 (retrospectively registered 11/1/2023).

**Supplementary Information:**

The online version contains supplementary material available at 10.1186/s44167-023-00018-4.

## Background

Physical activity (PA) improves general wellbeing through better physical and mental health [1] however more than one in four adults (27.5%) do not meet physical activity guidelines [2]. Physical inactivity is a global public health challenge associated with a substantial disease burden and global healthcare costs of $53.8 billion [3, 4], accounting for 6–10% of major non-communicable diseases (e.g., diabetes, heart disease, cancers, etc.) [5] and 7.2% of all-cause mortality [3]. With only 15 min of MVPA per day, a 14% reduction in all-cause mortality risk is postulated [6] and if PA guidelines are met, this increases to a greater than 50% reduction [7]. Development of effective and scalable interventions that target increased physical activity are needed.

Pedometers, wearable devices that measure and display daily step count in real-time, have been considered an effective tool for fostering self-regulatory behavioral skills to increase PA [1, 8–10]. They are affordable and readily available to consumers, making them a less time-consuming and resource-intensive approach than other strategies [11, 12]. Meta-analyses of pedometer based interventions have shown a medium positive effect (Cohen’s d = 0.72, 95% confidence interval (CI) 0.56, 0.88) on PA in adults [9] with increases of around 26% from baseline, which are equivalent to 2000–2500 steps per day [8]. A meta-analysis of 57 pedometer studies found that the positive effects on daily steps [mean difference (MD) (95% CI) 1126 (787, 1466) steps per day] lasted beyond the immediate short term (i.e. up to four-months) and were somewhat sustained [MD (95% CI) 434 (191, 676) steps per day] in the long term (i.e., up to four-years) [1].

Technological advancements have led to the evolution of traditional pedometers [1, 13, 14]. Activity trackers (e.g., Fitbits) are wearables that integrate additional features such as: long-term data storage and access; enhanced wear versatility (hip, pocket, bra and wrist); measurements of PA intensity and duration, and time spent sedentary; additional sensors and associated physiological data (e.g., sleep, heart rate and caloric expenditure); and real-time feedback [14–16]. Associated apps also provide features such as social networking in online communities, goal setting (e.g., 10,000 steps/day, stand time), prompts and rewards [14–16]. Their additional features (e.g., automated goal setting and social networking) have shown positive effects on PA [17–20], which combined with self-monitoring, may result in increased effectiveness compared to a standard pedometer [21]. Alternatively, the increased cost and complexity of wearable activity trackers may make them less user friendly and attractive [1, 22]. The prevalence of research on the effectiveness of activity trackers in activity interventions has increased markedly over the last decade. There is evidence of the preliminary effectiveness of activity trackers as self-monitoring tools to increase PA behaviors, with reported positive effects [11, 12, 23–25] and with mean differences of up to 950 (95% CI 476, 1425) steps per day [12] compared with no intervention.

Although there is substantial, if varying, evidence to suggest both pedometers and activity trackers are effective for increasing PA, research is unclear on whether the additional features in activity trackers enhance their effects on PA beyond those of traditional pedometers. A meta-regression by Chaudhry, Wahlich [1] estimated that the short-term (< 4 month) effect of studies using pedometer-based interventions was 834 steps (95% CI 126, 1542) more than studies using activity trackers or smartphone apps. This suggests activity trackers may be inferior to traditional pedometers in effecting a change in ambulatory PA measured by daily step count. However, indirectly comparing two interventions in such a meta-analysis can be limited if the study populations and other features of interventions (such as educational material) are markedly different. Few studies have directly compared the effectiveness of activity trackers and pedometers [25, 26]. In the 16-week randomised trial with inactive, post-menopausal women, there was no clear statistical difference between the effectiveness of a Fitbit ONE and a traditional pedometer on PA levels, with sedentary behavior not being assessed [25]. The participants assigned activity trackers increased their step count by an average of 427 steps per day, and increased MVPA by 7.0 min per day compared to those in the pedometer group by the end of the intervention, but confidence intervals were broad enough to be consistent with no difference or a slight advantage to pedometers. Similarly, another pilot trial found that pedometers and activity trackers improved MVPA [26]. While the magnitude of change was greater in the activity tracker group, statistical significance was not explored and the activity tracker group had substantially lower baseline MVPA, The evidence is therefore mixed: results of a meta-regression suggest pedometers may be superior to activity trackers but is limited by pooling heterogenous studies of different interventions and populations, and a direct comparison suggests activity trackers may be no different, or slightly superior, to pedometers but is limited by lack of statistical clarity. Further data are required on the relative effectiveness of activity trackers and traditional pedometers.

The aim of this exploratory study was to compare the effectiveness of two activity trackers (Fitbit ONE and Jawbone UP) and a traditional pedometer (Digi-Walker SW200) as self-monitoring tools for increasing MVPA and step count, and reducing sedentary behavior, in previously inactive adults aged 18–65 years. Given the additional features of activity trackers, it was hypothesised that both the activity tracker interventions would be more effective at increasing MVPA and step count, and decreasing sedentary behavior, than the traditional pedometer.

## Methods

### Study design and participants

This study used a one-group-per-condition design. The study was approved by The University of Queensland Ethics Committee (2014000766) and was conducted in adherence with the National Statement on Ethical Conduct in Human Research [27]. The study was conducted between July and December 2014.

Potential participants were recruited from three campuses of a large metropolitan university via convenience sampling. Posters, email advertisements and recruitment presentations were used to recruit participants. Potential participants were informed about the purpose of the study, which was to determine if activity trackers were more effective than pedometers for increasing physical activity in inactive adults. Individuals were asked to indicate their interest via email to the research team and were then forwarded a study information sheet and consent form by email. To be eligible for inclusion, participants had to meet the following criteria: employed full-time in a desk-based role at either site, be aged between 18 and 65 years, own or have access to a smart phone, not be pregnant or planning pregnancy and doing less than 150 min of MVPA per week (i.e., below current PA recommendations), as measured by the Active Australia survey [2, 28]. Eligibility was screened by a telephone call with a member of the research team.

Eligible participants were then invited to an individual appointment at the university with a researcher to complete their initial assessment. Participants were grouped into one of three groups according to their recruitment campus to attempt to control for social factors. An independent researcher randomly allocated each group to receive either the ONE, UP or PED. Data analyses were conducted by a researcher blinded to group allocation by deidentifying groups with pseudonyms until data analysis completion. Although participants were unable to be blinded to group allocation, they were blinded to the study’s hypothesis. Participants received a $50 voucher at the completion of the 16-week study period.

### Intervention

The primary active intervention component was providing the participants with a device (and the associated App where applicable). Beyond this, there was intentionally no additional active intervention components to approximate the real-world scenario of purchasing an activity tracker or pedometer ‘off the shelf’. After randomisation, participants received their ONE, UP or PED for a concurrent eight-week intervention phase. In the first week, a one-hour group-based information session was conducted to orient the participants to their devices and to provide technical support for set up. A second one-hour group-based face-to-face support session was conducted in week six to troubleshoot device issues and technical barriers to use. Participants received no education or encouragement to be more active or less sedentary in these sessions. To monitor progress, each participant in the ONE and UP groups provided the researchers with their online device account details. The PED group were provided with a written activity log to record their daily step count, which they provided to the research team via email or in hard copy.

### Devices

The Digi-Walker SW200 (Yamax, Bridgnorth, England, United Kingdom) is a clip-on pedometer designed for continuous wear on the waist, excluding water-based activities (https://www.yamax.co.uk/yamax-pedometers/sw200-digi-walker/). A coiled spring-suspended lever arm in an internal sensor mechanism measures steps and immediate feedback on cumulative steps is provided through a small display on the device. It has a three-year battery life, and step count must be manually reset at the day’s conclusion. The waist worn Digi-Walker has good validity (r_s_ = 0.56) and reliability (r_s_ = 0.79) for step count in free-living walking and improves with increasing walking speed [29].

The FitBit One (Fitbit Inc, San Francisco, California, United States of America, 2014) is a small non-waterproof, clip-on activity tracker that contains an altimeter and a triaxial accelerometer and is designed for continuous 24-hour wear, excluding water-based activities, either on the waist, pocket or bra (https://www.fitbit.com/pl/shop/one). It measures active time, daily steps, floors climbed, miles walked, caloric expenditure and sleep duration and quality. A small display provides immediate feedback on these data, or data can be viewed by syncing the device with the Fitbit app on a smart phone or web-based platform via Bluetooth. The device has a ten-day battery life and can store seven-days of minute-by-minute detailed data and the preceding 23 daily summarises. When hip worn, the Fitbit ONE has excellent validity (CCC = 0.97-1.00) and reliability (ICC = 0.99) for step count in treadmill walking [30].

The Jawbone UP (Jawbone, San Francisco, California, United States of America, 2014) is a wrist-worn, waterproof activity tracker that contains a triaxial accelerometer and is designed for continuous 24-hour wear [15]. It measures daily steps, active time, idle time, caloric expenditure and sleep duration and quality. When the user is ‘idle’ for a period of time, it vibrates to remind the user to move. With no display on the device, data are displayed by syncing the device to the Jawbone app on a smart phone via the built-in headphone jack. It has a ten-day battery life and can store nine-months of detailed data. The Jawbone UP demonstrates strong validity (r = 0.97) and reliability (ICC = 0.97) for step count in free-living conditions [31].

Both activity trackers can link to an online public community and tools through their respective smartphone apps [15]. These can provide additional feedback through graphing performance, adjusting goals, tracking additional health outcomes (e.g. weight or blood pressure) and interactions with other participants.

### Demographic and anthropometric data

A paper based demographic questionnaire was used to collect participants’ age and gender. Anthropometric data were measured according to the International Society for the Advancement of Kinanthropometry. Weight was measured using portable scales (MS-3200, Charder, Taiwan) and height by a portable stadiometer (217 stadiometer, SECA, Germany). Measurements were taken twice and the average was recorded.

### Outcome measures

#### Accelerometry

Minutes per day of MVPA, sedentary behavior and daily step count were assessed at baseline and four-, eight- and 16-weeks (8 weeks after the conclusion of the 8-week intervention) using accelerometry (Actigraph GT3X+, Pensacola, Florida, United States of America). The Actigraph is a small, reliable [32] and valid [33], non-invasive device that measures bodily movement [34, 35]. Participants were encouraged to continuously wear the device on their right hip using a belt for seven-days except when sleeping or engaging in water-based activities or during contact sports. During monitoring periods, participants recorded wake and sleep times and device on/off times, including the reason for removal if longer than 10 min. A 30 Hz sampling frequency was used to initialise the accelerometer and prior to analysis, raw data from the .gt3x file were converted to 30-second epochs. Data were included if participants provided a minimum of four valid days (defined as a minimum wear time of eight-hours per day), including one weekend day, of accelerometry data [36]. Non-wear time was defined as 60-minutes or more of consecutive zero counts per minute (CPM) with a spike tolerance of two-minutes [37]. ActiLife (version 6; ActiGraph, Pensacola, Florida, United States of America) was used to determine minutes per day of sedentary behavior and MVPA from the vertical axis data for each valid day with the following cut points: sedentary behavior < 100 CPM [38] and MVPA ≥ 2020 CPM [39]. Within-instrument processing of the number of cycles in the accelerometer signal was used to estimate step counts [40].

### Statistical analysis

Demographic characteristics were analysed using descriptive statistics. To evaluate intervention effects, data from all eligible participants with valid baseline accelerometry data were included and analysed on an intention-to-treat basis using last observation carried forward (LOCF) to account for missing data [41]. Waterfall plots were used to visualise the changes in the outcomes from baseline to 16-weeks for each individual participant. Linear mixed-effect regression models, one model for each outcome measure, were performed on the change in each of the outcome measures relative to baseline (i.e., for each participant, the value at week 0 was subtracted from the values at 4, 8 and 16 weeks). A group by time interaction term estimated the fixed effect of group allocation on the rate of change in the outcome measures over time. Participants were treated as a random effect, having a random slope with respect to time. A fixed intercept of zero was specified, representing the baseline value. The slopes (regression coefficients) of the group by time interaction (i.e., the slope over time for each group) were compared using one-way ANOVA, with Satterthwaite degrees of freedom approximation. Post-hoc comparisons between each of the two activity tracker groups (ONE and UP) against the PED group were conducted using Dunnett’s test. Alpha was set at 0.05.

Data analysis was performed using *R* (version 4.1.2, The R Foundation for Statistical Computing, Vienna, Austria), using the ‘lme4’ package (version 1.1–30) [42] for regression modelling, ‘lmerTest’ package (version 3.1-3) [43] for subsequent ANOVA tests and ‘emmeans’ package (version 1.7.5) for post-hoc Dunnett tests [44].

No sample size calculation or power analysis was performed as this study was exploratory in nature.

## Results

### Participant characteristics

**Fig. 1:**
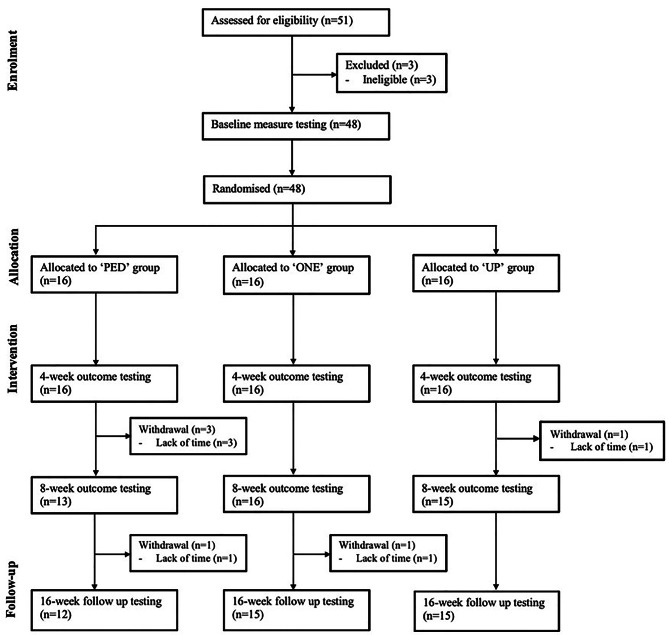
CONSORT flow diagram of participant recruitment, enrolment and progression through the study. *Note*: PED = Digi-Walker SW200, ONE = Fitbit ONE, UP = Jawbone UP

Fifty-one participants were screened for eligibility. Forty-eight participants (average age of 40.5 years and 83% female) met the eligibility criteria and were enrolled in the study (Fig. 1 presents the study flow diagram; Table 1 presents sociodemographic and anthropometric characteristics of participants). Following baseline testing, participants were allocated into one of the three groups based on their campus of recruitment: ‘PED’ (n = 16), ‘ONE’ (n = 16) and ‘UP’ (n = 16). The study’s overall retention rate was 87.5% with six [‘PED’ (n = 4), ‘ONE’ (n = 1), ‘UP’ (n = 1)] participants withdrawing during the 16-week period due to “lack of time”.

**Table 1:** Baseline sociodemographic and anthropometric characteristics of participants (N = 48)

Characteristics	Whole sample (n = 48)	‘PED’ group (n = 16)	‘ONE’ group (n = 16)	‘UP’ group (n = 16)
Age (years)	40.56 (9.31)	39.14 (9.63)	40.21 (10.51)	42.31 (8.05)
Height (cm)	165.22 (8.20)	162.51 (8.85)	164.26 (7.60)	168.88 (7.18)
Weight (kg)	73.77 (21.13)	74.71 (29.61)	72.58 (17.48)	74.03 (14.62)
BMI (kg/m^2^)	26.85 (6.39)	27.80 (8.35)	26.92 (6.47)	25.82 (3.76)
Female, n (%)	40 (83.3)	12 (75)	14 (87.5)	14 (87.5)
Step count (steps/day)	6876 (1606)	6557 (2111)	7156 (1496)	6853 (1281)
MVPA (minutes/day)	26 (12)	23 (13)	26 (12)	29 (10)
Sedentary behavior (hours/day)	9.9 (1.1)	10.3 (1.0)	9.3 (1.2)	10.1 (1.0)

*Note*: Data are presented as mean (standard deviation) unless otherwise specified

PED = Digi-Walker SW200, ONE = Fitbit ONE, UP = Jawbone UP, BMI = body mass index, SD = standard deviation, MVPA = moderate-vigorous physical activity

### Outcome measures

Data from 46 out of 48 participants [‘PED’ (n = 15), ‘ONE’ (n = 15), ‘UP’ (n = 16)] were included in the analyses, with two excluded due to invalid baseline accelerometry data. Valid data were available for 96% of all participants at baseline, 79% at four-weeks, 81% at eight-weeks and 83% at 16-weeks. Taken together, loss of data due to wear time criteria and attrition resulted in 20% of missing values from the outcome measure dataset. For those with valid data, mean wear time was high [‘group’ mean (standard deviation; SD) ‘PED’ 13.80 (1.48), ‘ONE’ 13.15 (1.25), ‘UP’ 14.10 (1.12) hours per day].

At baseline, one-way ANOVA did not indicate any statistically significant difference between groups for step count (F[2, 39] = 0.46, *p* = 0.64, η^2^ = 0.02), MVPA (F[2, 39] = 0.85, *p* = 0.44, η^2^ = 0.04) or sedentary behavior (F[2, 39] = 3.17, *p* = 0.053, η^2^ = 0.14).

**Fig. 2:**
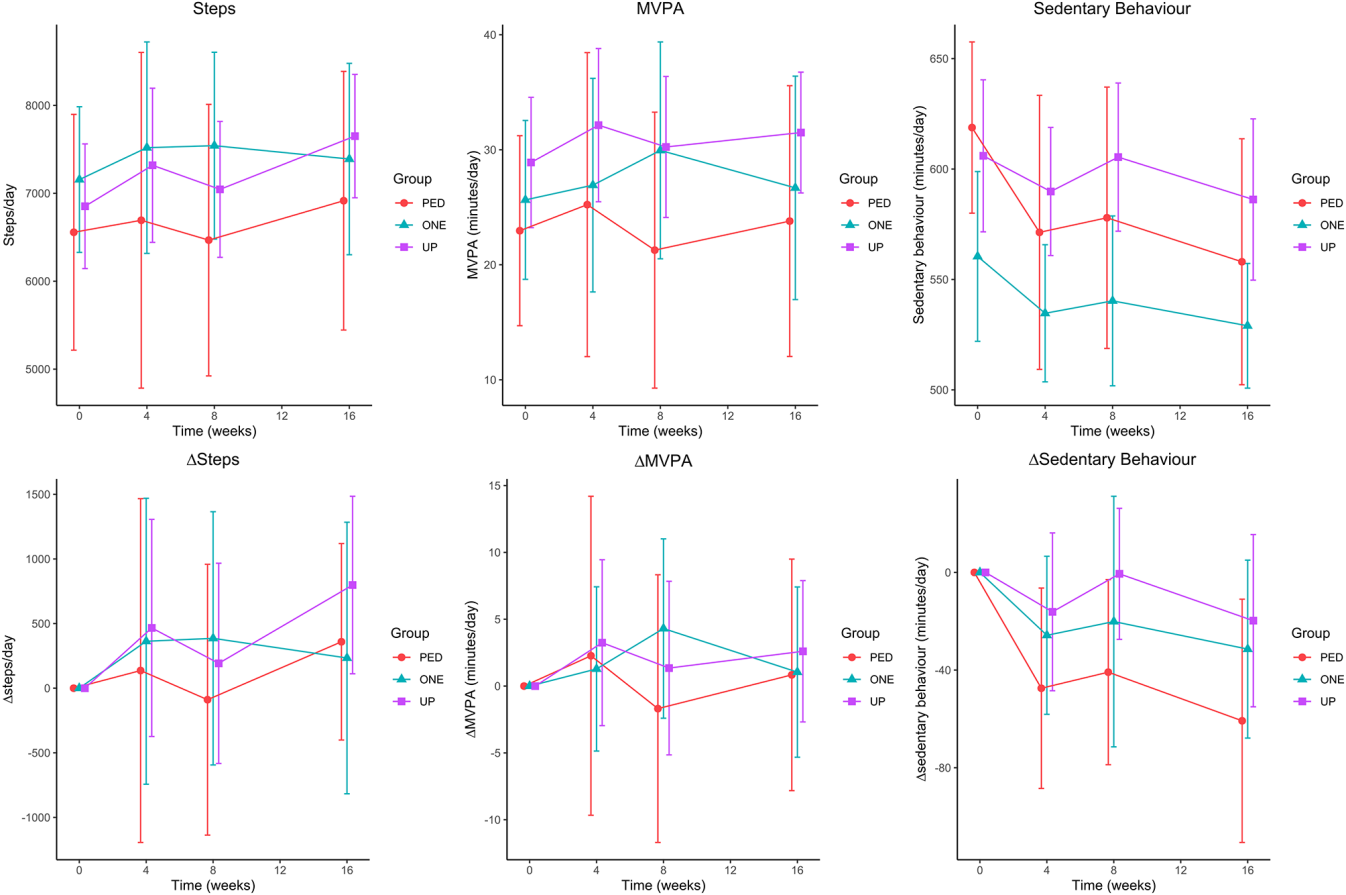
Mean (and 95% confidence interval) for daily steps, MVPA and sedentary behaviour at 0, 4, 8 and 16 weeks in the three groups, changes (Δ) from baseline. *Note*: PED = Digi-Walker SW200, ONE = Fitbit ONE, UP = Jawbone UP, MVPA = moderate to vigorous physical activity

**Fig. 3:**
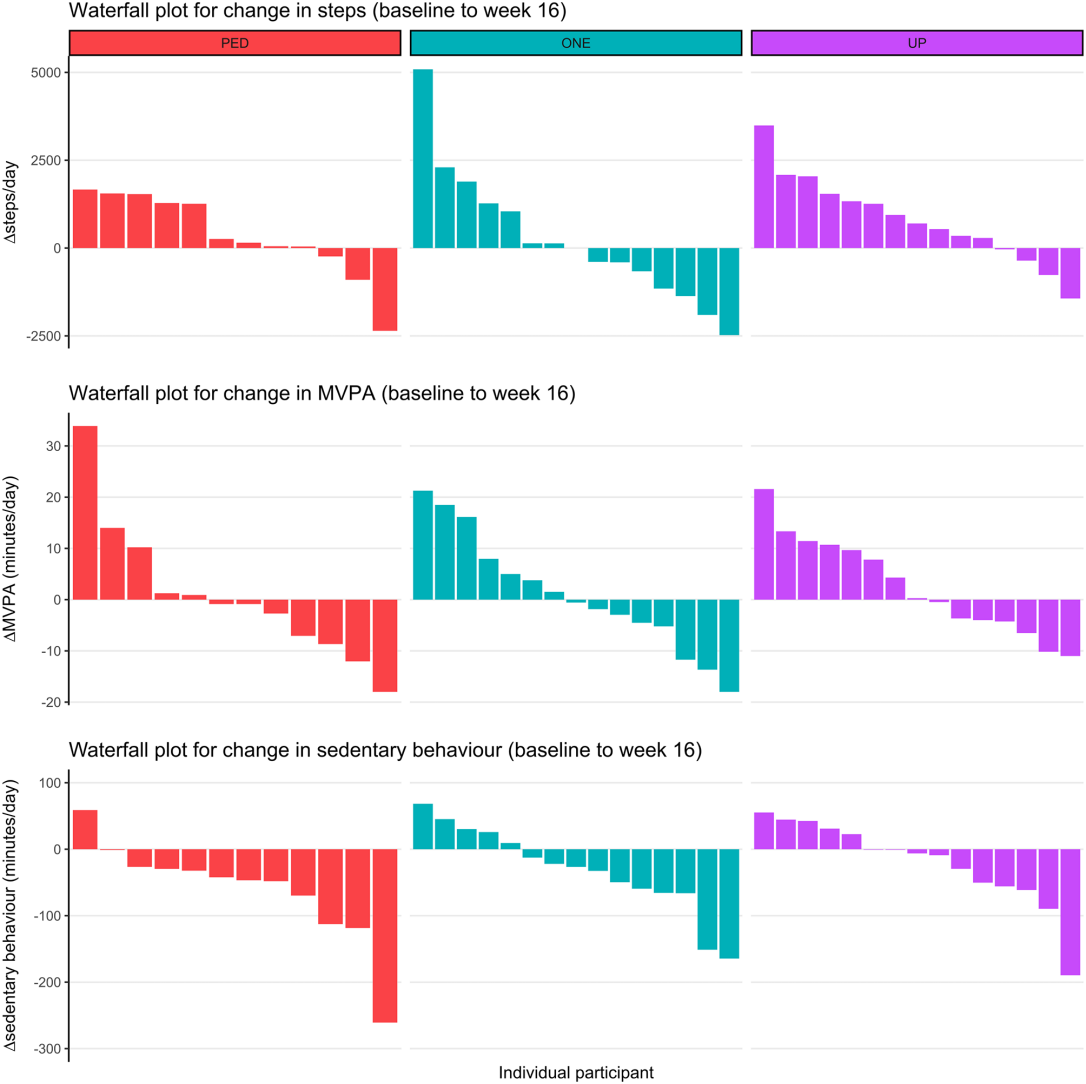
Waterfall plots for individual changes between baseline and week 16 for the outcomes of daily steps, MVPA and sedentary behaviour in each group. *Note*: PED = Digi-Walker SW200, ONE = Fitbit ONE, UP = Jawbone UP, MVPA = moderate to vigorous physical activity

Figure 2 illustrates mean (and 95% CI) raw scores and change scores (relative to baseline) for step count, MVPA and sedentary behavior across all assessment time points (baseline to 16-weeks), for each group. Individual changes in step count, MVPA and sedentary behavior from baseline to 16-weeks are presented as waterfall plots in Fig. 3.

**Fig. 4:**
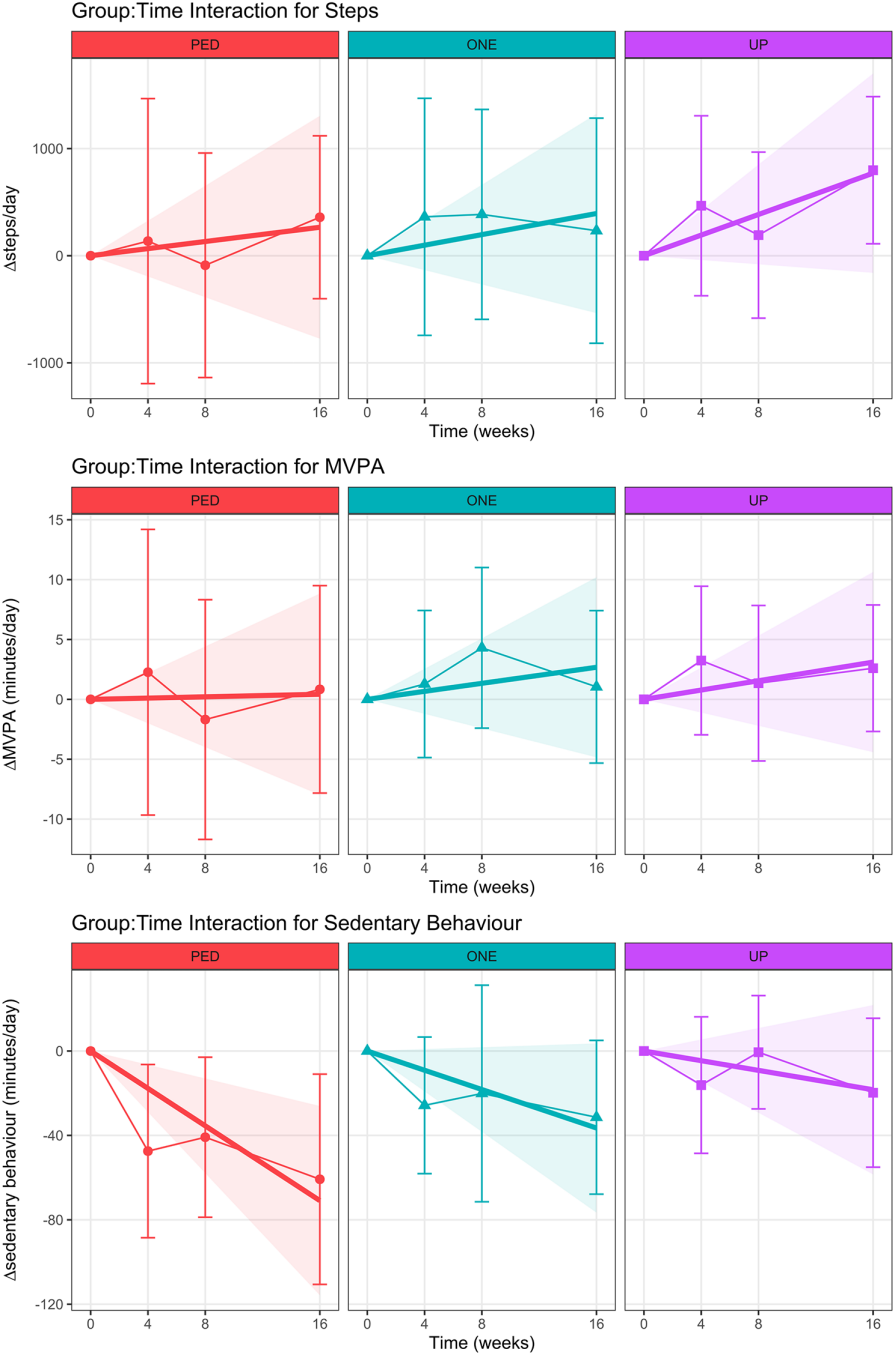
Group-by-time interactions from the linear mixed-effect regression analysis for changes from baseline for daily steps, MVPA and sedentary behaviour. The slope of the line represents the estimated values (based on the linear regression) for the mean effect for that group over time, with shaded areas representing the 95% confidence interval for that line. Points represent the observed group mean at each time point and error bars represent the 95% confidence interval about that mean. *Note*: PED = Digi-Walker SW200, ONE = Fitbit ONE, UP = Jawbone UP, MVPA = moderate to vigorous physical activity

Figure 4 illustrates the slopes of the group-by-time interaction effects derived from the linear mixed-effect regression modelling of change scores (i.e., difference from baseline) for each outcome variable, along with the observed group-level means at each time point (as in Fig. 2). Table 2 outlines the results of this regression analysis, as well as the one-way ANOVAs of group:time interaction effects and post-hoc Dunnett’s tests of change from baseline scores in each of the activity tracker groups (ONE and UP) compared to the PED group.

**Table 2:** Results from regression analysis, ANOVA and post-hoc tests of change from baseline scores

Outcome	Group	Group:Time Interaction (95% CI)	Estimated change at 16 weeks (95% CI)	One-way ANOVA	Post-hoc Dunnett’s test of Group:Time Interaction (95% CI) vs. PED	Estimated difference in change at 16 weeks (95% CI) vs. PED
**Daily step count (steps/day)**	PED	16.6 (− 49.4, 82.6)	266 (− 791, 1322)	F(3,42) = 1.21, p = 0.32		
	ONE	24.6 (− 34.4, 83.7)	394 (− 551, 1339)		8.0 (− 93.6, 109.6)	129 (− 1497, 1754)
	UP	48.1 (− 10.9,107.2)	770 (− 174, 1715)		31.5 (− 70.1, 133.1)	504 (− 1120, 2130)
**MVPA (minutes/day)**	PED	0.03 (− 0.51, 0.56)	0.43 (− 8.12, 8.97)	F(3,42) = 0.39, p = 0.76		
	ONE	0.17 (− 0.31,0.64)	2.68 (− 4.96, 10.32)		0.14 (− 0.68, 0.96)	2.3 (− 10.9, 15.4)
	UP	0.19 (− 0.28, 0.67)	3.11 (− 4.53, 10.75)		0.17 (− 0.65, 0.99)	2.7 (− 10.5, 15.8)
**Sedentary behaviour (minutes/day)**	PED	−4.4 (− 7.3, − 1.6)	−71 (− 116, − 25)	F(3,42) = 4.60, p = 0.007		
	ONE	−2.3 (− 4.8, 0.3)	−37 (− 77, 4)		2.2 (− 2.2, 6.5)	34 (− 35, 104)
	UP	−1.1 (− 3.7, 1.4)	−18 (− 59, 22)		3.3 (− 1.1, 7.7)	53 (− 18, 123)

PED = Digi-Walker SW200, ONE = Fitbit ONE, UP = Jawbone UP, BMI = body mass index, CI = confidence interval, ANOVA = analysis of variance, MVPA = moderate-vigorous physical activity

A one-way ANOVA of the group-by-time interaction effects derived from the linear mixed-effect regression modelling of change scores for steps did not find a statistically significant difference between the groups for rate of change in step count (F[3, 42] = 1.21, *p* = 0.32). Post-hoc comparisons using Dunnett’s test between the two activity tracker groups (ONE and UP) and the PED group, multiplied by 16-weeks to calculate the estimated difference in the effect by the end of the study period, estimated the ONE group increased step count by 129 steps/day ([95% CI − 1497, 1754], *p* = 0.97) more than the PED group and the UP group increased by 504 steps/day ([95% CI − 1120, 2130], *p* = 0.69) more than the PED group.

A one-way ANOVA did not find a statistically significant difference between the groups for rate of change in MVPA (F[3, 42] = 0.39, *p* = 0.76). Based on post-hoc comparisons, the difference in the change in MVPA using model estimates at 16-weeks was 2.3 min per day ([95% CI − 10.9, 15.4], *p* = 0.88) higher in the ONE group than in PED group and 2.7 min per day ([95% CI − 10.5, 15.8], *p* = 0.84) higher in the UP group than the PED group.

Over the 16-week study period, sedentary behavior in the ONE group changed at a mean rate of − 2.3 min/day/week [95% CI − 4.8, 0.3] while sedentary behavior in the UP group changed at a rate of − 1.1 min/day/week [95% CI − 3.7, 1.4]. This was a slower rate of change than that seen in the PED group, which averaged a change in sedentary time of − 4.4 min/day/week [95% CI − 7.3, − 1.6]. A statistically significant difference across the groups for this rate of change in sedentary behavior was detected by one-way ANOVA (F[3, 42] = 4.60, *p* = 0.007). Based on post-hoc comparisons, the PED group had a rate of decrease that was 2.2 min/day/week faster than ONE ([95% CI − 2.2, 6.5], *p* = 0.43) and 3.3 min/day/week faster than the UP group ([95% CI − 1.1, 7.7], *p* = 0.17), neither of which were statistically significant differences.

Additional File 1 includes graphs illustrating the individual random effects for the linear mixed-effect regression modelling of change scores for the three outcome variables.

## Discussion

This study compared the effectiveness of two activity trackers (Fitbit ONE and Jawbone UP) and the Digi-Walker SW200, as self-monitoring tools for improving MVPA, reducing sedentary behavior and increasing step count in inactive adults using a pragmatic, one-group per-condition randomised controlled trial design. At 16-weeks, all three groups demonstrated an increase in steps and MVPA, and a decrease in time spent in sedentary behaviour, however there was substantial individual variation in these outcomes and the hypothesis was unable to be confirmed.

In this study, the differences between the activity tracker groups and the pedometer group for changes in step count (from baseline) are similar in both magnitude and width of confidence intervals to those reported in a randomized controlled trial [25]. That study also indicated a non-significant difference between the activity tracker (Fitbit) and pedometer of 427 steps per day, favoring the Fitbit, after a 16-week intervention (N = 25 and N = 26 per group, respectively). This is of the same order as the non-significant increase of 504 more steps per day (95% CI − 1120, 2130) in the UP group than the PED group reported in the current study. While both studies are limited by small sample sizes, a recent meta-analysis estimated that a 475 steps (95% CI 190, 784) difference was significant in those with activity trackers compared those without [11]. However, when comparing activity trackers and pedometers, the findings from a meta-regression by Chaudry et al. [1] estimate a significant difference of -834 steps (95% CI − 1542, −126) in favour of pedometers over activity trackers, in the short-term (< 4 month). The researchers concluded that pedometer-based interventions were superior for increasing steps compared to activity trackers. This may reflect the limitations of indirect comparisons between different studies in a meta-regression, rather than randomizing the same sample population to make a direct comparison between two interventions or performing a meta-analysis of direct comparisons from randomised controlled trials. However, it is also important to emphasize that all existing estimates for the difference between activity trackers and pedometers have confidence intervals that would be compatible with pedometers being slightly superior to activity trackers (i.e. 100 to 1000 steps more by the end of the intervention) for increasing steps. Further research with much larger sample sizes is required to gain more precise estimates of the difference between these two types of devices.

The current study is the first to directly compare the effects of activity trackers (ONE and UP) and a pedometer (PED) on sedentary behavior in free-living conditions, in addition to physical activity. At the end of the intervention there was a decrease in time spent in sedentary behavior of 71 min/day (− 116, − 25) in the PED group, which was 34 (-35, 104) min/day more than in the ONE group and 53 (-18, 123) minutes/day more than in the UP groups. The width of the confidence intervals, however, indicated no significant differences between groups on post-hoc comparisons in the effect on sedentary time when using the two activity trackers and the pedometer. A meta-analysis of 14 randomized controlled trials compared multi-factorial PA interventions that included traditional pedometers with control interventions [45] and found a significant small benefit of pedometer-based interventions, with a Cohen’s d − 0.20 (95% CI − 0.33, − 0.07) for change in sedentary behavior, equivalent to − 23 min per day (95% CI − 38, −8), which is consistent with the reduction in sedentary behavior seen in the current study. It is important to also note though, that findings from the current study may have been impacted by baseline group differences in sedentary behaviour which approached significance.

The estimates derived from this study and prior research [1, 25] suggest that the comparative effects of activity trackers and pedometers are inconclusive, however direct comparisons are limited and future research is needed. If future research can confirm at least the equivalence of these devices, then selection of wearable devices for promoting PA and reducing sedentary behavior may be based on user preference and resources. This would provide greater flexibility and accessibility, as well as increasing the ability to individually tailor interventions which may leverage the high amount of individual variation seen in these studies.

The current study has several strengths. This study’s findings provide valuable insight into the real-world utility of activity trackers and pedometers as consumers predominately purchase them with no additional education, indicating that they likely need to form part of a multifaceted PA intervention. Assigning all participants from each site to a single intervention allowed the study to control for various social factors, such as group cohesion, that may occur in the real-world as people typically connect with others using the same device. Also, the study attempted to reduce the types of bias common to behavioral interventions by blinding the group allocations during data analysis, blinding participants to the study’s hypothesis and standardising assessment procedures [46, 47]. Despite attrition, imputation of missing values using LOCF permitted an ITT analysis, reducing the bias that could be introduced by non-adherence, providing a more realistic estimate of likely adherence when implemented into the general population [48–50].

Several limitations must also be acknowledged. Despite the above-mentioned reasoning for allocating the devices by group, this study is limited by having only one group per intervention condition. As such, the intra-group correlation could not be estimated, and lacking any external estimates of this correlation, the analysis could only be performed under the assumption that this correlation was zero and treating observations as if each participant was independent of others [51]. However, a non-zero correlation is likely due to shared exposure to workplace environment and the social influence of participants over others. For instance, participants who are highly motivated to increase their step count may influence their co-workers to take a walk as a group during break times. Indeed, a goal of grouping participants by worksite was to facilitate this same sort of interaction between participants, which was then assumed to be absent for data analysis. Therefore, the results of this study must be interpreted with caution.

Limitations of this study must also be acknowledged. The small sample size, while highlighting considerable heterogeneity in responses, limits the power to detect groups differences if there are any, and limits generalisability. Further, withdrawals rates were higher in the PED group (n = 4) compared to the ONE (n = 1) and UP groups (n = 1). This may be due to the pedometer being less engaging and interactive compared to the activity trackers. Our convenience sampling resulted in a sample of predominately overweight, middle-aged women, which prevents generalisation of the findings for men, and other age and BMI categories. Although the self-reported measures commonly overestimate PA [52], participants may have under reported PA in their screening questionnaire to meet the study’s inclusion criteria (doing less than 150 min of MVPA per week) as baseline accelerometry indicated total MVPA averaged 26 min per day (~ 182 min per week). Although similar to the baseline total MVPA values for the study population of a prior randomised controlled trial [25], this may have limited the participants’ capacity for improvement, underestimating the effects of each intervention. While the fact that the models of activity trackers used in this study are no longer available must be acknowledged as a limitation, the principles of the hypothesis being tested are still relevant and have the capacity to inform future research. Lastly, the study was conducted predominantly throughout spring in Brisbane, Australia. The findings may therefore be impacted by a seasonal effect, given that given that MVPA has been shown to increase in warmer months[53]. Future research could use longer follow up to explore the impact of these seasonal effects combined with the use of activity trackers.

## Conclusion

This exploratory study found considerable variation in responses to wearing a pedometer or an activity tracker tracker in terms of increasing step counts and MVPA and decreasing sedentary behavior in inactive adults. Although, on average, all three groups demonstrated increases in daily steps and MVPA and decreases in sedentary behavior, changes over time and differences between groups were largely insignificant. Future research should investigate with more statistical certainty the difference, or equivalence, in the effectiveness of activity trackers and pedometers with adequately powered sample sizes, as well as further explore the individual variation and the potential for individually tailored support to maximise health behaviour change.

## Electronic supplementary material

Below is the link to the electronic supplementary material.

Supplementary Material 1

## Data Availability

The dataset generated and/or analysed during the current study are available from the corresponding author on reasonable request.

## Abbreviations

PA: Physical activity
MVPA: Moderate to vigorous physical activity
CCC: Concordance correlation coefficient
CI: Confidence interval
MD: Mean difference
ONE: Fitbit ONE
UP: Jawbone UP
PED: Digi-Walker SW200
SB: Sedentary behavior
CPM: Counts per minute
LOCF: Last observation carried forward
ITT: Intention to treat
ANOVA: Analysis of variance
N: Sample sie
BMI: Body mass index
