# Supplementary material for: TRACK & ACT: a pragmatic randomised controlled trial exploring the comparative effectiveness of pedometers and activity trackers for changing physical activity and sedentary behaviour in inactive individuals
Source: J Act Sedentary Sleep Behav. 2023 May 1;2:12. doi: 10.1186/s44167-023-00018-4 (PMC11960358; doi:10.1186/s44167-023-00018-4)
Supplement: Supplementary file 1 — Supplementary Material 1 [file 44167_2023_18_MOESM1_ESM.docx]

**Additional File 1**

(a)


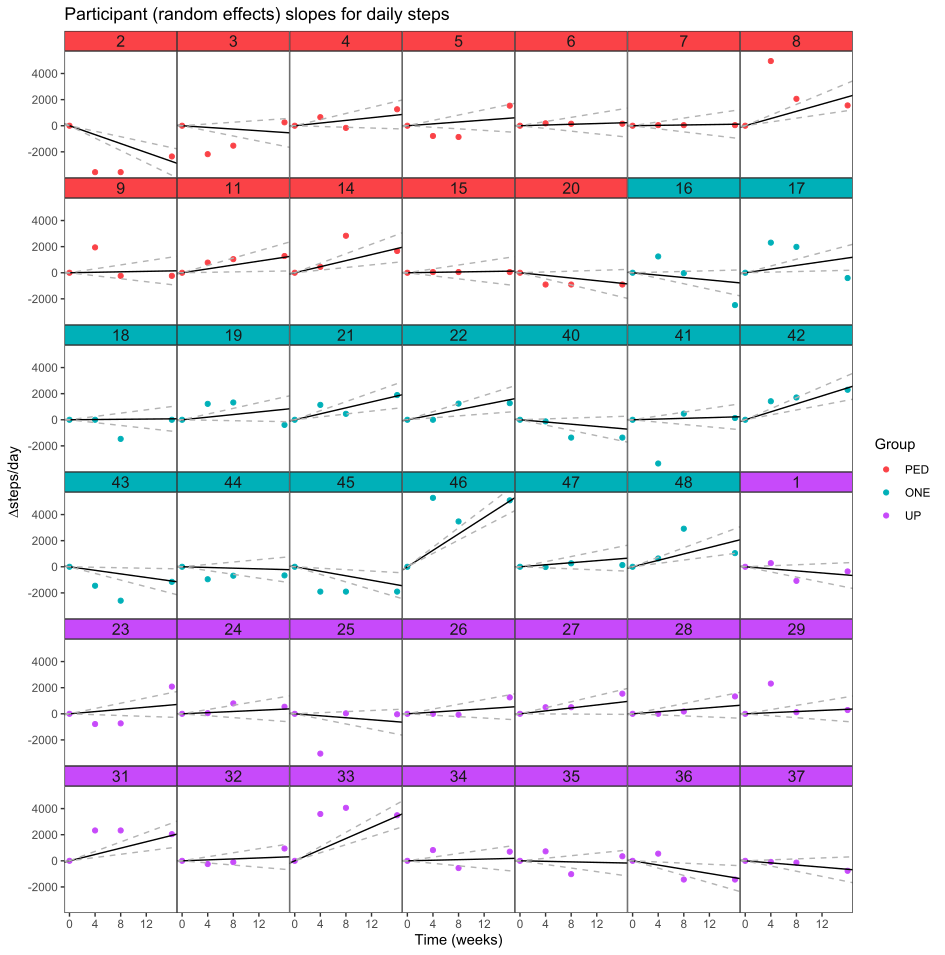


(b)


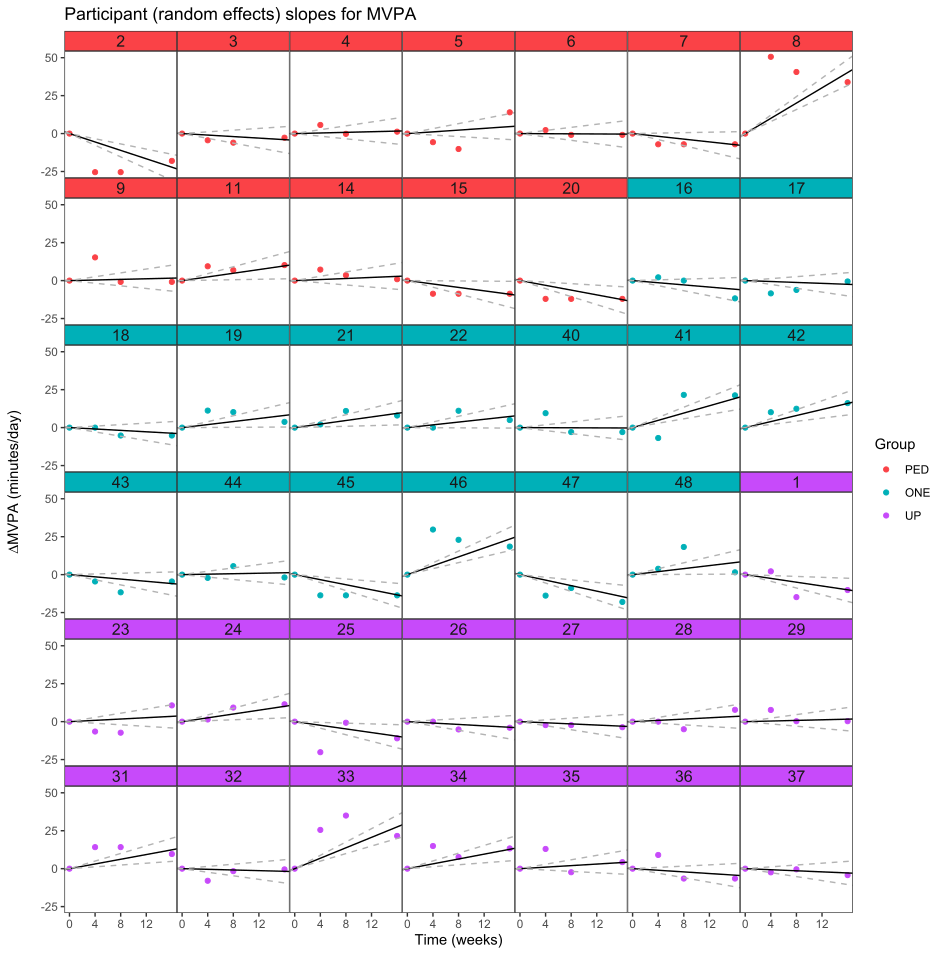


(c)


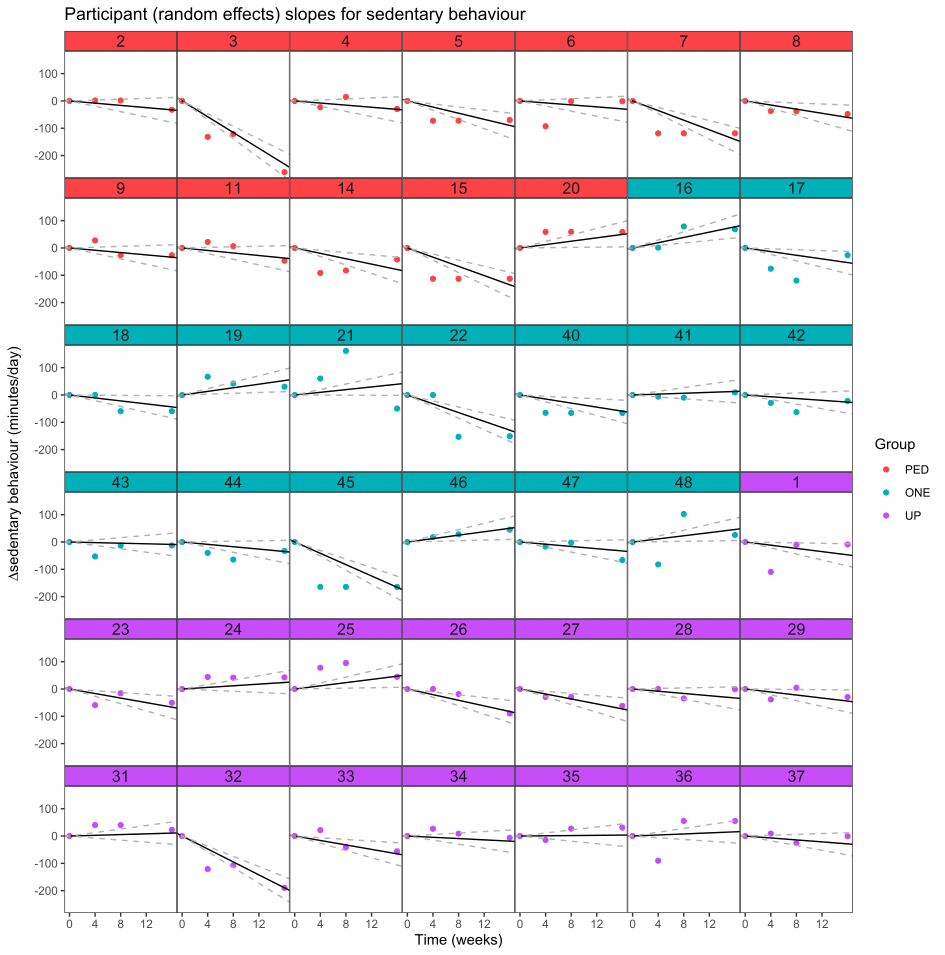


**Figure 1.** Lattice plot showing the individual-level slopes for (a) steps, (b) MVPA and (c) sedentary behaviour from the linear mixed-effect regression. Points represent the individual change from in baseline. Solid line shows the sum of the group-by-time slope and the individual random effect. Dotted lines represent the sum of the upper or lower bounds of the 95% confidence interval for the group-by-time slope and the individual random effect. *Note:* PED = Digi-Walker SW200, ONE = Fitbit ONE, UP = Jawbone UP, MVPA = moderate to vigorous physical activity.
